# Using patient input to develop item banks to measure quality-of-life impact of vitreous floaters

**DOI:** 10.1136/bmjophth-2025-002658

**Published:** 2026-05-07

**Authors:** Jarinne Woudstra-de Jong, Sonia Manning-Charalampidou, Tirza Voogt-den Hertog, Johannes R Vingerling, Jan J Busschbach, Konrad Pesudovs

**Affiliations:** 1 Research and Innovation, Eye Hospital Rotterdam, Rotterdam, The Netherlands; 2 Erasmus Medical Center, Department of Psychiatry, Section of Medical Psychology and Psychotherapy, Erasmus Universiteit Rotterdam, Rotterdam, The Netherlands; 3 Vitreoretinal Surgery, Eye Hospital Rotterdam, Rotterdam, The Netherlands; 4 Erasmus Medical Center, Department of Ophthalmology, Erasmus Universiteit Rotterdam, Rotterdam, The Netherlands; 5 Erasmus Medical Center, Section of Medical Psychology and Psychotherapy, Erasmus Universiteit Rotterdam, Rotterdam, The Netherlands; 6 School of Optometry and Vision Science, University of New South Wales, Sydney, New South Wales, Australia

**Keywords:** Vitreous, Retina, Treatment Surgery, Posterior Chamber

## Abstract

**Aims:**

To develop item banks for a patient-reported outcome measure (PROM) specific to vitreous floaters using input from patients. We report on the content generation and item refinement, and compare the content of the newly-developed PROM with published literature.

**Methods:**

Potential PROM items (questions) were identified from two sources: 20 existing floaters-specific PROMs and two qualitative studies in patients with floaters. This initial item pool was evaluated with binning (grouping) and winnowing (reduction) to group the PROM items into quality-of-life domains. Patients with floaters provided feedback on the pilot PROM item banks in cognitive interviews. The Dutch PROM item banks were translated into English, Arabic and Turkish. Each step was guided by an expert panel consensus.

**Results:**

The initial item pool with potential PROM items consisted of 921 items. After three rounds of binning and winnowing, the item pool was reduced to 272 items. After 10 cognitive interviews, 19 items were changed, 8 were deleted and 8 were added. The final PROM item pool consists of 272 items across 12 item banks (quality-of-life domains): visual symptoms, ocular symptoms, general symptoms, activity limitations, driving, mobility issues, health concerns, economic impact, emotional well-being, social well-being, inconvenience and coping.

**Conclusion:**

The new floaters-specific PROM item banks included all 81 items from previous PROMs, supplemented with additional quality-of-life issues reported by people experiencing floaters (70.2%). The largest quality-of-life domains were ‘health concerns’ and ‘inconvenience’, emphasising the disease burden of experiencing vitreous floaters and patients’ information needs. Item banking allows clinicians and researchers to choose what domains and items to use in their measurement. Additional quality-of-life issues identified in previously unstudied populations can be added and calibrated with the existing items. Future studies using the item banks can compare the quality-of-life impact of different clinical subgroups, and control for important confounders.

**WHAT IS ALREADY KNOWN ON THIS TOPIC**

- Existing patient-reported outcome measures (PROMs) for vitreous floaters are limited in scope and lack comprehensive coverage of the quality-of-life impact reported by patients.

**WHAT THIS STUDY ADDS**

- This study systematically developed and refined item banks that comprehensively represent the lived experience of vitreous floaters across 12 quality-of-life domains. 70.2% of the 272 items have not been measured before.

**HOW THIS STUDY MIGHT AFFECT RESEARCH, PRACTICE OR POLICY**

- The new PROM item banks provide a standardised, patient-centred framework for assessing the impact of vitreous floaters. They can support outcome measurement in clinical research and inform patient-focused care strategies.

## Introduction

 Many people experience vitreous floaters, but not all suffer from them. The impact of floaters on quality of life differs per individual,^1^,^3^ and is said to depend on factors such as work, age and myopic status.^4^ A subset of people suffering from floaters visits one or even multiple ophthalmologists for possible treatment.^5^ It is difficult for ophthalmologists to gauge the severity of floaters based on visual acuity or any other clinical indicator. Having a valid instrument that measures quality-of-life impact can help in decision-making by measuring the impact of floaters and outcomes after possible treatment. Such instruments are called patient-reported outcome measures (PROMs), self-reported questionnaires that are a valuation of the personal experience of someone experiencing floaters. Unfortunately, current PROMs used for vitreous floaters are suboptimal; they were developed without comprehensive patient input, and have poor measurement properties and limited content.^1^ Thereby, the patient voice has been under-represented in clinical research.

For many ophthalmic conditions, PROM item banks have been developed.^6^,^9^ An item bank is a representative, optimally informative and efficient set of items (questions) that measures a quality-of-life domain, such as activity limitations or emotional well-being. Traditional PROM questionnaires require respondents to fill out all items, even those that may not be relevant to them, which may result in high respondent burden, frustration and therefore possible data quality issues. Item banks permit selection of items in short-form versions or computer adaptive testing, where the length of the questionnaire is optimised in real time during the administration. Item banks can also easily be updated. To develop a valid and relevant set of PROM items, patient consultation is crucial.^10 11^ The purpose of our research was to develop the content of floaters-specific PROM item banks together with patients. In this study we report on the content generation and item selection.

## Materials and methods

This study follows the methods of the ‘Eye-tem bank project’^12^ to develop PROM item banks for patients with vitreous floaters, and guidelines developed by the US Food and Drug Administration (FDA) and the COnsensus-based Standards for the selection of health Measurement Instruments (COSMIN).^10 11^ We established the content of the item banks in four phases: (1) content identification, (2) ‘binning and winnowing’, (3) cognitive interviews and (4) translation. The study adhered to the tenets of the Declaration of Helsinki. All participants signed informed consent.

### Content identification

We used different sources to identify potential PROM items. From a literature review, we identified existing floaters-specific PROMs and published qualitative studies.^1^ We extracted all items from 20 current disease-specific PROMs developed for patients with floaters. We found one qualitative study investigating the psychological experience of 11 Italian patients with floaters, and extracted all reported quality-of-life issues.^2^ In addition, we conducted in-depth interviews with 44 Dutch patients on the impact of floaters and potential treatment on their quality of life.^3^ All quality-of-life issues that were identified from qualitative studies were rephrased into potential PROM items by two authors (JW-dJ and SM-C), and formatted towards standardised ‘item stems’ per quality-of-life domain, for example, ‘How much difficulty do you have*…’* for ‘Activity limitations’.

### Construction of questionnaire format

The Dutch item stems and response categories were formulated based on established English item stems and response categories in item banks for other eye conditions (Australia),^6^,^9^ other often-used Dutch questionnaires and expert opinion (item stems in online supplemental table A). Consistent with previous ophthalmological item banks, we used four to five response categories per item. We formulated three scales for each of the three symptoms domains (visual symptoms, ocular symptoms and general symptoms), that is, frequency, severity and nuisance.^13^ We also translated the English questionnaire instructions into Dutch. During the cognitive interviews, we specifically inquired about comprehensibility of the Dutch item stems, response categories and questionnaire instructions.

### Item evaluation (binning and winnowing)

After we identified the list of all potential PROM items (initial item pool), we selected and classified the items under previously-established ophthalmological quality-of-life domains, based on their colloquial meaning. Previous work on quality of life and PROMs in ophthalmology has identified the following 12 domains: visual symptoms, ocular symptoms, general symptoms, activity limitations, driving, mobility issues, health concerns, economic impact, emotional well-being, social well-being, inconvenience and coping.^6^,^9^ We evaluated any item that did not seem to fit under a previously defined domain to verify if a new domain is necessary to fully determine quality of life in vitreous floaters. The classification and refinement of items followed the method of ‘binning and winnowing’, consistent with the methods of the Patient-Reported Outcomes Measurement Information System (PROMIS) group.^14^ Binning is a systematic process to organise the item list, where items with the same or very similar meaning are grouped together under one quality-of-life domain. Winnowing is a systematic process to reduce the item list into a minimally representative list, following a list of four criteria: (1) inconsistency with the domain definition, (2) similarity to another item, (3) narrow content without wider applicability and (4) being confusing or unclear. Two authors (JW-dJ and SM-C) employed successive cycles of binning and winnowing; they consulted another author (KP) in the case of indecisiveness.

### Expert opinion

The content development process was mainly performed by two authors (JW-dJ and SM-C). During all development phases, we consulted expert opinions (JJB is an expert on quality-of-life measurement, KP is an optometrist and expert on quality-of-life measurement in ophthalmic diseases and JRV and SM-C are vitreoretinal specialists).

### Pre-testing: cognitive interviews

The 12 predefined quality-of-life dimensions formed the basis for the pilot item banks. Items were presented per item bank to patients with floaters to elicit feedback on the instrument.^14^,^16^ There are different justifications for sample size in cognitive interviewing.^17^,^19^ In our study, we chose a sample size of 10, based on previous studies using cognitive interviews to optimise PROMs and patient surveys in ophthalmology and different guidelines for cognitive debriefing interviews.^1214^,^16^ We aimed to include a variety of participants with different characteristics to get a broad overview of possible problems with interpretation. Another goal was testing the clarity of using the Dutch language, as previous Eye-tem bank studies were done in Australia and Asia.

#### Inclusion and exclusion criteria for cognitive interviews

To assure sufficient variance in the sample, we selected patients with different ages, genders, levels of education and stages of treatment (no treatment indicated, on waiting list for vitrectomy or after vitrectomy). We included patients with floaters in one or both eyes, as diagnosed by an ophthalmologist, that were willing and able to participate in an interview. Patients were excluded for declining participation, cognitive impairment (dementia, acquired brain injury, intellectual disability), age younger than 18 years and any visually significant comorbid ophthalmic disorder, for example, endophthalmitis, uveitis, (diabetic) retinopathy, exudative age-related macular degeneration or visually significant glaucoma. We also excluded patients whose floaters were caused by vitreous haemorrhage or uveitis. We did not exclude patients with comorbid or previous cataracts. There was no overlap in participants of the content identification interviews and cognitive interviews.

#### Recruitment of participants for cognitive interviews

Participants were recruited between June and August 2023 from the Rotterdam Eye Hospital, an eye centre in the Netherlands providing secondary and tertiary care. JW-dJ and SM-C screened the outpatient vitreoretinal clinical lists to identify potential participants. Eligible patients were approached after the clinical consult and were given information about the study. People that were interested in the study were given a participant information flyer and consent form. After participation in the cognitive interview, participants received a gift voucher of €25 or could opt to donate the money to retina research.

#### Data collection during cognitive interviews

The pilot item banks were administered face-to-face to patients with floaters by JW-dJ, to elicit feedback on the instrument and to assess whether the instructions, items and response categories were well understood. Participants were specifically asked to give feedback on the language, comprehensibility, relevance and coverage across each quality-of-life domain. Any problematic items and comments were noted. Three of the authors evaluated all patient feedback (JW-dJ, SM-C and TV-dH). Based on the patients’ feedback, items were added, deleted or rephrased.

### Translation procedure

The final item banks were translated from Dutch into Arabic, English and Turkish by Wilkens B.V. (Powerling group), a Dutch translation agency (ISO certifications 9001, 17100, 13485). All translators were native speakers of the target language (Arabic, English or Turkish) and specialised in medical translations. The linguistic translation procedure adhered to international guidelines.^20^,^24^ The aim of the translation process was to obtain items in Arabic, English and Turkish that are equivalent to the Dutch items. First, the original Dutch item pool was translated into the target language by two independent translators (forward translation). The two forward translations were reviewed and synthesised by the translation agency to create a single reconciled version per target language. A third independent translator translated the reconciled version back into Dutch (backward translation). The back translation was compared against the original Dutch item pool by the research team (JW-dJ, SM-C and TV-dH). Any potential errors were identified and rectified based on discussions with the translators. The final Dutch, Arabic, English and Turkish versions were compared against each other and any discrepancies were fixed. This resulted in four language versions of the floaters-specific item banks: Dutch, Arabic, English and Turkish.

### Patient and public involvement

The Client Board of the Rotterdam Eye Hospital reviewed and advised on the study protocol during the grant application process. The list of potential PROM items was informed by 44 patient interviews. Also, 10 patients gave feedback on the pilot item pool (see Results: Cognitive interviews). The results were shared to study participants during yearly newsletters and during the Vitreoretinal Patient Day organised by the study team on 2 October 2025.

## Results

### Content identification

**Figure 1:**
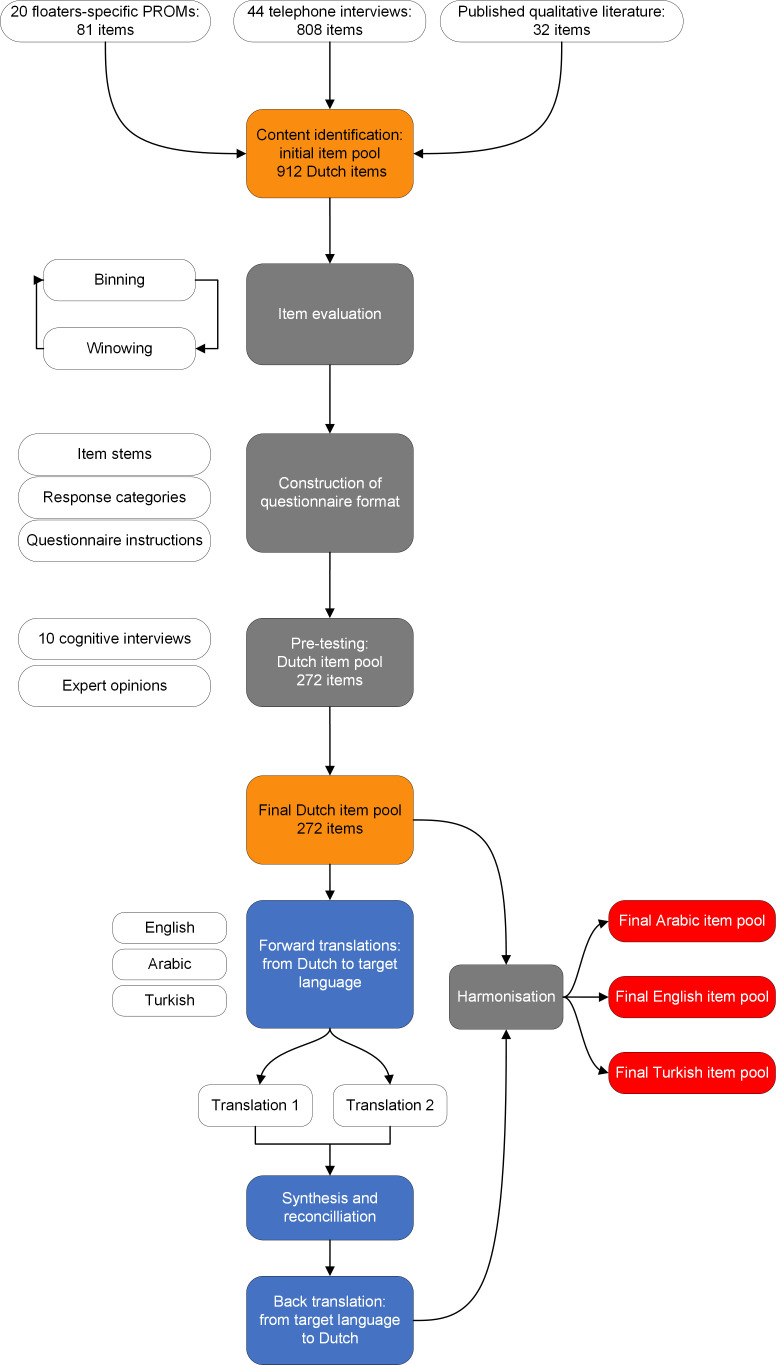
Content development for vitreous floaters-specific item banks. PROMs, patient-reported outcome measures.

The initial item pool with potential PROM items consisted of 921 items (table 1, figure 1): 81 items from 20 disease-specific PROMs developed for patients with floaters^1^ (list of PROMs is shown in online supplemental table B); 32 items from an explorative qualitative study with 11 patients^2^; and 808 items from 44 in-depth interviews.^3^ All items identified in the qualitative studies and current PROMs were again identified during the 44 in-depth interviews, with additional items that were not previously reported in the scientific literature. The 921 items could all be classified into the pre-conceptualised domains: visual symptoms, ocular symptoms, general symptoms, activity limitations, driving, mobility issues, health concerns, economic impact, emotional well-being, social well-being, inconvenience and coping. A list of quality-of-life domains and related subthemes can be found in online supplemental table C. There was discussion about a floaters-specific domain related to problems with different lighting conditions; however, based on previous experiences with development of other ophthalmic item banks, these items were rephrased into ‘Visual symptoms’, for example, ‘how often do you experience vitreous floaters in bright light or sunlight?’. Of the 921 initial items, most items grouped into ‘Visual symptoms’ (243 items), ‘Health concerns’ (128) and ‘Emotional wellbeing’ (122)—these were the largest initial quality-of-life domains. The smallest initial domains were ‘General symptoms’ (31 items), ‘Economic impact’ (31) and ‘Mobility’ (16).

**Table 1:** Content development for the vitreous floaters-specific item banks with number of items per item bank

Quality-of-life domains (item banks)	Items from existing PROMs^1^	Items from exploratory qualitative study^2^	Items from telephone interviews^3^	Items in initial potential item pool	Items retained after binning and winnowing process	Final item pool after cognitive interviews
Activity limitations	33	2	63	99	31	30
Convenience	2	0	69	71	37	37
Coping	0	6	82	88	30	28
Driving	1	0	10	11	5	5
Economic impact	3	2	29	31	14	14
Emotional well-being	8	6	108	122	33	32
General symptoms	0	0	31	31	11	14
Health concerns	1	5	122	128	45	43
Mobility issues	3	0	13	16	7	7
Ocular symptoms	0	1	42	43	22	21
Social well-being	1	4	33	38	10	10
Visual symptoms	29	8	206	243	27	31
Total items	81	32	808	921	272	272

PROM, patient-reported outcome measure.

### Binning and winnowing

There were many conceptually similar items in the initial item pool. The largest reduction of items was the ‘Visual symptoms’ domains; for example, we could group together many different wordings to describe floaters (spots, circles, dots, flies, patches, specks, etc) and their movement patterns (dancing, flying, floating, swirling, etc) into the item ‘seeing moving spots’. Also, we only kept negatively-phrased emotions under the ‘Emotional wellbeing’ domain, to prevent possible multidimensionality from positively-phrased emotions. In three rounds of ‘binning and winnowing’, we removed 649 items, resulting in a pilot item pool of 272 items (table 2).

**Table 2:** Examples from the binning and winnowing phase

Original item from the interviews	Action during binning and winnowing
How much difficulty do you have driving in the dark? In the rain? In the mist?	Binning – Binned together, ie, How much difficulty do you have driving in poor visibility (in the dark, mist or rain)?
How often do you experience misty vision? Cloudy vision? Hazy vision? Vision as if you are looking through a veil?	Winnowing – Kept the most representative item (ie, experiencing cloudy vision), removed the other items.
How often do you feel hopeful? How often do you feel hopeless?	Winnowing – Removed all positively-phrased emotional concepts (eg, hopeful) and kept negative counterpart (eg, hopeless).
How much trouble is not doing everything you want to do?	Removed – Item was unclear and too general.
How much difficulty do you have reading the menu in a dimly lit restaurant?	Removed – Item was too specific.
How often do you experience stinging pain in your eye?	Removed – Item was similar to another item (ie, How often do you experience stinging in your eye?).

### Cognitive interviews

This pilot item pool was subjected to 10 cognitive interviews (sample characteristics are shown in online supplemental table D), after which the wording of 19 items was changed, 8 items were deleted and 8 items were added (table 3). The participants did not have any issues with the Dutch item stems. Some participants opted to change the response categories in the symptom domains, for example, ‘always’ (Dutch: ‘altijd’) instead of ‘very often’ (Dutch: ‘vaak’); however, we decided to stick with ‘very often’ to stay in line with the English item stems used in other ophthalmic item banks. The questionnaire instructions were mostly clear. We only decided to add reminders throughout the questionnaire that respondents should answer based on their current status (eg, for the inconvenience domain: how much trouble is it now?), not hypothetically (how much trouble would it be if that happened to me?) or retrospectively (how much trouble has it been before?). We placed ‘Please answer on how you are doing at the moment’ at the start of every domain.

**Table 3:** Examples from cognitive interview phase

Original item from the interviews	Action after cognitive interviews
How often do you see floaters?	Added ‘How often do you see a dark floater in the middle of your vision?’, as several participants described that floaters progressed from transient and see-through to dark and staying in the middle of their vision.
How often do you feel dispirited? How often do you feel dejected?	Binned items together, that is, ‘How often do you feel discouraged?’
How much trouble is everything taking a lot of effort?	Changed into ‘How much trouble is everything taking more effort?’
How much do you try everything to be able to see well?	Removed – Item was unclear.
How severe are your stiff muscles from lying still during surgery or because of posturing?	Split into two items, that is, ‘How severe are your stiff muscles from lying still during surgery’ and ‘How severe are your stiff muscles from posturing?’

### Vitreous floaters-specific item banks

After these refinements, the final vitreous floaters-specific PROM item banks consist of 272 items across 12 quality-of-life domains: visual symptoms (31 items), ocular symptoms (21 items), general symptoms (14 items), activity limitations (30 items), driving (5 items), mobility issues (7 items), health concerns (43 items), economic impact (14 items), emotional well-being (32 items), social well-being (10 items), convenience (37 items) and coping (28 items). Examples of items for each domain are listed in online supplemental table E. The full item list is available on reasonable request.

## Discussion

In this paper we have reported on the development of vitreous floaters-specific patient-reported outcome item banks. The item banks include 272 items across 12 quality-of-life domains. This new PROM covers all 81 items that exist in previous floaters-specific PROMs.^1^ We have built on these existing items in the literature and included 191 additional items (70.2%) that are brought forward by patients with floaters, coming from qualitative studies with patients with floaters.^2 3^ We then checked the content and clarity of the items in cognitive interviews with 10 patients and made changes according to their feedback. Item generation is seen as incomplete without patient consultation, also according to the FDA guidelines for PROM development.^10^ Our approach, which aligns with these guidelines, enabled us to develop PROM items that are both important and relevant to individuals with vitreous floaters.

Interestingly, the item banks with the most items are the domains ‘Health concerns’ and ‘Convenience’. Thus our results suggest that reassuring patients in relation to their concerns and providing clear information, as well as awareness of the many inconveniences caused by floaters, might be useful in patient–doctor consultation. In comparison, ‘Activity limitations’ and ‘Emotional wellbeing’ are the largest domains in other ophthalmic item banks, such as diabetic retinopathy, retinal disease, glaucoma and refractive error.^6^,^9^ This deviation highlights the importance of administering disease-specific items in people experiencing floaters, that target specific issues that are relevant to this specific eye condition.

The item banks have been translated into English, Arabic and Turkish, which are the main minority languages in the Netherlands. This translation process was purely a linguistic translation, not a cultural adaptation. Future studies should provide cultural validations for the translations with pretesting (cognitive debriefing) with English-speaking, Arabic-speaking and Turkish-speaking individuals, in collaboration with bilingual medical professionals and the developers of the item banks.

Recently, the Vitreous Macula Retina (VMR) Institute published a validation study of the Vitreous Floaters Functional Questionnaire (VFFQ-23).^25^ The content of the VFFQ-23 is floaters-specific and developed with patient input, with evidence of test–retest reliability and correlations with clinical measures. The VFFQ-23 was based on 51 items developed by a floaters patient association with expert advisers, covering six dimensions: disabling effects, constant awareness, worry about persistence, bodily focus, no escape or help and fear of deterioration. All 51 items are included or resemble items in our 272-item bank (18.8%). One main advantage of using item banks over static PROMs is their flexibility, as clinicians and researchers can choose which specific items to include in their measurement, for instance through using validated short forms or computer adaptive testing applications. Both short forms and computer adaptive testing can considerably reduce administration burden, by reducing the number of items needed to reach a reliable estimate. Also, item banks can be updated: any relevant items we missed can be added to the appropriate item bank and calibrated.

A major strength of our study is that the item banks are developed based on an extensive literature search and patient input. Our aim was to develop the content together with patients, to ensure that the item banks are relevant, comprehensive and comprehensible. The possibility exists that additional quality-of-life issues are relevant for patients in specific populations that have not been researched previously by us or other research groups. We are currently conducting interviews with floaters sufferers from different countries and age groups, including young adults, to corroborate our results. Any additional quality-of-life issues that are identified can be added to the appropriate item bank and calibrated with the existing items. Likewise, additional cognitive interviews in different populations may raise additional problems around comprehensibility, comprehensiveness and relevance that we did not identify in this study. Following Beaty and Willis,^15^ the strongest claim from cognitive interviews that could be made is that no problems have been discovered or have not been discovered yet. Notably, in our current ongoing validation study using the item banks, we encourage participants to share any comments relating to the comprehensibility, comprehensiveness and relevance of the item banks and will adjust the items and/or instructions if need be.

The quality-of-life impact of vitreous floaters can vary when comparing different subgroups, such as people from different clinical stages and treatment phases, different cultures and countries or different age groups. We deliberately interviewed people from a heterogeneous background to get a broad view of the possible impact that vitreous floaters and treatment can have. Notably, the goal of our qualitative studies was not to stratify these experiences by subgroups. A goal in current and future quantitative research using the item banks as outcome measures will be to compare the quality-of-life impact of floaters and possible treatment between different subgroups. These future quantitative studies should also take clinical confounders into account, such as the presence or history of cataracts, laterality of eye floaters and involvement of the dominant eye, which may meaningfully influence symptom severity, functional impairment and other patient-reported outcomes. These are important confounders that future studies have to control for when assessing patient-reported outcomes.

To integrate the patient voice in research, we recommend the use of PROMs that have been developed with patient input, such as our newly-developed item banks. Our vitreous floaters-specific item banks consist of 272 items across 12 quality-of-life domains that cover the full breadth of the possible quality-of-life impact of vitreous floaters. The item banks can be used as a PROM to answer highly relevant clinical questions, such as differences in the quality-of-life impact between different subgroups, correlations with clinical assessments and specific treatment effects after different types of treatment. Using all item banks enables measurement across a broad spectrum of affected life areas that have not been adequately measured before, in comparison to clinical measures and previous PROMs that often focus on symptoms and functioning only. Optionally, clinicians and researchers can choose which domains and items to include in their measurement, for instance based on expected treatment results related to a specific quality-of-life domain. Also, our item banks include items that are only relevant to a specific subgroup of patients; for example, the item ‘concerns about complications from treatment’ is only relevant for patients that are considering surgery or other types of treatment. Because we have developed item banks instead of a fixed-form questionnaire, it is possible to skip items that are not relevant during PROM administration while still producing a reliable score. Our research group is currently collecting data from patients with floaters to calibrate the item banks and provide evidence for psychometric quality.

In conclusion, vitreous floaters affect an individual’s life in many aspects. Our newly-developed floaters-specific PROM item banks have the potential to provide a broad measurement of affected quality-of-life domains. The item banks can be used as a grading of disease severity, as well as a measurement of treatment effectiveness. The item banks can provide new insight into the impact of vitreous floaters and treatment that has not been measured previously and will allow for computer-adaptive testing in the future.

## Supplementary material

10.1136/bmjophth-2025-002658 online supplemental file 1

## Data Availability

Data sharing not applicable as no datasets generated and/or analysed for this study.
