# Supplementary material for: Using patient input to develop item banks to measure quality-of-life impact of vitreous floaters
Source: BMJ Open Ophthalmol. 2026 May 7;11(2):e002658. doi: 10.1136/bmjophth-2025-002658 (PMC13157721; doi:10.1136/bmjophth-2025-002658)
Supplement: online supplemental file 1 [file bmjophth-11-2-s001.pdf]

Supplementary materials for "*Using patient input to develop item banks to measure quality-of-life impact of vitreous floaters*"

1. Table A. Dutch item stems and response categories, translated from English
2. Table B. Number of items extracted from existing patient-reported outcome instruments developed for people experiencing vitreous floaters.
3. Table C. Overview of quality-of-life domains and related sub-themes from interviews with patients with floaters (N = 44).
4. Table D. Socio-demographic and clinical characteristics of the participants of the cognitive interviews (*N* = 10)
5. Table E. Examples of patient-reported outcome item development in various quality-of-life domains based on 44 interviews with patients with vitreous floaters

Table A. Dutch item stems and response categories, translated from English

Item bank (domain)	English item stem and answer categories[1]	Dutch translated item stem and answer categories
Symptoms – Frequency (visual/ocular/general)	How often do you experience…? (very often, quite often, occasionally, never)	Hoe vaak …? (vaak, regelmatig, soms, nooit)
Symptoms – Severity (visual/ocular/general)	How severe is/are …? (severe, moderate, mild, not at all)	Hoe erg is/zijn …? (heel erg, matig, mild, niet erg)
Symptoms – Nuisance (visual/ocular/general)	How much of a problem is/are…? (a lot, quite a bit, a little, none)	Hoe hinderlijk is/zijn …? (heel hinderlijk, best hinderlijk, een beetje hinderlijk, niet hinderlijk)
Activity limitations<br>Driving<br>Mobility issues	How much difficulty do you have …? (unable to do because of my vision, a lot, quite a bit, a little, none)	Hoeveel moeite heeft u met …? (kan ik niet vanwege mijn zicht, veel moeite, best wat, een beetje, geen moeite)
Health concerns<br>Economic impact	How concerned are you about …? (extremely, a lot, a moderate amount, a little bit, not at all)	Hoeveel zorgen heeft u over/dat …? (extreem veel, veel, best wat, een beetje, geen)
Emotional wellbeing	How often do you …? (all of the time, most of the time, some of the time, a little of the time, none of the time)	Hoe vaak …? (altijd, vaak, soms, zelden, nooit)
Social wellbeing	How much of problem do you have …? (unable to do because of my vision, a lot, quite a bit, a little, none)	Hoe lastig is …? (extreem lastig / dit kan ik niet vanwege mijn zicht, heel lastig, best lastig, een beetje lastig, niet lastig)
Inconvenience	How much trouble is …? (extremely, quite a lot, a moderate amount, a little bit, none)	Hoe vervelend is …? (extreem vervelend, heel vervelend, best vervelend, een beetje vervelend, niet vervelend)
Coping	How much do you cope by …? (extremely, quite a bit, a moderate amount, a little bit, not at all)	Hoeveel …? (extreem veel, heel veel, best veel, een beetje, dit doe ik niet)

Note: For most of the domains respondents can answer ‘This issue is not relevant to me / Don’t do this task’ (Dutch: ‘Dit is niet relevant voor mij / Ik doe dit nooit’)

Table B. Number of items extracted from existing patient-reported outcome measurement (PROM) instruments developed for people experiencing vitreous floaters.

Patient reported outcome instruments	Study: First author (year)	Items
Frankfurt Floater Questionnaire	Koch (2014)[2]	22
Floater Disturbance Questionnaire	Ankamah (2021)[3]	4
Floater Prevalence Survey	Webb (2013)[4]	2
Modified National Eye Institute Visual Functioning Questionnaire	De Nie (2013)[5]	26
Short Floater Questionnaire	Tassignon (2016)[6]	4
Visual Function Questionnaire	Schiff (2000)[7]	12
Visual Quality of Life Questionnaire	Hahn (2018)[8]	14
Vitreous Floaters Symptom Questionnaire 1	Kim (2017)[9]	7
Vitreous Floaters Symptom Questionnaire 2	Lin (2022)[10]	13
Vitreous Floaters Symptom Questionnaire 3	Osmancevic (2019)[11]	32
unnamed instrument	Inouye (2021)	3
unnamed instrument	Mason(2014), Lin (2016)[12,13]	8
unnamed instrument	Rao (2022)[14]	3
unnamed instrument	Schulz-Key (2011)[15]	8
unnamed instrument	Shah (2020), Ludwig (2021), Delaney (2002) [16–18]	6
unnamed instrument	Traill (2016)[19]	5
unnamed instrument	Tsai (1993)[20]	1
unnamed instrument	von Fricken (2009)[21]	7
unnamed instrument	Zeydanli (2020)[22]	6
single PROM items: ratings of the severity of floaters, visual disturbance, inconvenience caused by floaters, or discomfort during surgery	Bessa (2019),[23] Cañote (2020),[24] Lam (2017),[25] Lin (2022),[10] Ludwig (2021),[16] Luo (2018),[26] Ma (2019),[27] Nunes (2021),[28] Singh (2020),[29] Souza (2020),[30] Sun (2019),[31] Trujillo-Sanchez (2018),[32] Waseem (2021)[33]	13
**Total PROM items**		**81**

Table C. Overview of quality-of-life domains and related sub-themes from interviews with patients with floaters (N = 44).

Quality-of-life domain	Sub-theme
Activity limitations	Distance vision
	Driving
	Exercise / Sports
	Finding things
	Free time activities
	Household tasks
	Near vision
	Reading and writing
	Using screens
	Visual activities, e.g. seeing color / contrast / depth
Convenience	Related to activity limitations
	Related to eye drops
	Related to floaters symptoms, e.g. being distracted
	Related to glasses / contact lenses
	Related to hospital visit, e.g. travel distance
	Related to posturing after surgery
	Related to waiting
Coping	Emotional regulation, e.g. acceptance
	Problem avoidance, e.g. ignoring the problem
	Problem solving, e.g. using sunglasses
	Seeking help, e.g. visiting healthcare provider
Economic impact	Other costs, e.g. new glasses
	Treatment-related costs
	Work
Emotional wellbeing	Anger
	Anxiety
	Changed self-image
	Depression
General symptoms	Fatigue
	Headaches
	Related to treatment, e.g. stiff muscles from posturing
	Sleep problems
Health concerns	Related to future vision impairment
	Related to getting correct and understandable information
	Related to risks and complications of treatment
	Related to safety, e.g. falling
	Related to treatment, e.g. getting surgery and anesthesia
Ocular symptoms	Discomfort
	Pain
	Red eyes
	Tired eyes / eye strain
Social wellbeing	Getting support
	Getting understanding
	Going out

	Socializing
Visual symptoms	Blurry vision
	Difference between image from both eyes
	Distorted vision
	Flashes
	Floaters
	Problems with lighting conditions, e.g. photosensitivity
	Related to tamponade, e.g. seeing gas bubble

Table D. Socio-demographic and clinical characteristics of the participants of the cognitive interviews (*N* = 10)

Variable	***Sample characteristics***
Age at time of interview	
Mean ± Standard deviation	64.6 ± 12.6 years
Range	38.7 to 84.9 years
Gender	
Female	3 patients (30%)
Residence	
Same city as hospital	2 patients (20%)
Same province as hospital	5 patients (50%)
Different province as hospital	3 patients (30%)
Marital status	
Married/Partnership	5 patients (50%)
Education level	
Primary or high school education	3 patients (30%)
Secondary vocational education	3 patients (30%)
Higher professional education or university	4 patients (40%)
Employment status	
Working	6 patients (60%)
Visual acuity (better eye), decimal (logMAR)	
Median	1.0 (0.00)
Range	0.8 to 1.0 (0.00 to 0.10)
Visual acuity (worse eye), decimal (logMAR)	
Median	0.8 (0.10)
Range	0.6 to 1.0 (0.00 to 0.22)
Time interval between floaters diagnosis and interview	
< 3 months	3 patients (30%)
3 months - 2 years	5 patients (50%)
> 2 years	2 patients (20%)
Laterality	
Bilateral	6 patients (60%)
Lens status of the affected eye	
Pseudophakic at time of diagnosis	5 eyes (25%)
Treatment	
Phacovitrectomy	6 surgeries (4 patients, 40%)
Vitrectomy	4 surgeries (3 patients, 30%)
No treatment	3 patients (30%)
Post-treatment complications	
High intraocular pressure (for < 3 months)	1 patients (10%)
Epiretinal membrane (asymptomatic)	1 patient (10%)
*Ocular comorbidity (visually inconsequential)**	
Yes	4 patients (40%)
*Medical comorbidities***	
Yes	6 patients (60%)

Footnote:
* Ocular comorbidities included high myopia (2), blepharitis (1), retinal defect (1) and strabismus (1).
** Medical comorbidities included hypertension (2), cancer (2), hypercholesterolemia (2), gout (1), and hyperthyroidism (1).

Table E. Examples of patient-reported outcome item development in various quality-of-life domains based on 44 interviews with patients with vitreous floaters[34]

**Quality-of-life domain**	**Subtheme**	**Quote from patient interviews**	**Patient-reported outcome item**
Activity limitations	Looking from side to side	*And I also have difficulty when I work with computer screens, so I have two screens in front of me. And when I look from the one screen to the other screen, then yes, I am bothered by the floaters.*	How much difficulty do you have looking from side to side?
Convenience	Having to pause during activities	*But with reading a book […], part of the page is missing. And that is so annoying, because you sit down. And you have to search [for the words] and move the book, when I am in the middle of the story. Yes, I have to stop reading then.*	How much trouble is having to stop what you are doing because the floaters get in the way of your vision?
Coping	Blinking	*You keep your eye very still and try to blink less. And well, then they [the floaters] float to the front of your vision again. So I have to blink for some time, and try to get them out of the way. So that is, well, quite annoying actually.*	How much do you cope with the floaters by blinking?
Driving	Checking mirrors	*"Quickly flashing my eyes is something I cannot do anymore, for instance in the care: quickly checking the mirrors or looking over my shoulder."*	How much difficulty do you have checking your mirrors while driving?
Economic impact	Working more slowly	*I repair microelectronics, which is quite detailed work, even when using magnification tools […]. It was hard, I was struggling, so I worked a lot more slowly, because I constantly had to make sure that the floaters were out of the way. Which is also why I went to the doctor with the request to fix [my vision].*	How concerned are you about working more slowly?

Table D. (continued)

Quality-of-life domain	Subtheme	Quote from patient interviews	Patient-reported outcome item
Emotional wellbeing	Feeling frightened	*We have a display with notifications in the bus, and I could not read it anymore […] When a floater blocks your vision, you see nothing for a short moment, and that scares you every time.*	How often do you feel frightened because the floaters suddenly appear in your vision?
General symptoms	Fatigue	*Especially in the evenings, I am tired, and I am just tired in my eyes, in my head. And I think: pff, I have had enough for today.*	How often do you experience / How severe is / How much of a problem is feeling tired?
Health concerns	Moving by mistake during surgery	*I chose for a general anesthetic. Yes, because then I cannot sneeze during the surgery, or at least, the chance of me moving is a lot smaller. […] You don't want the doctor to slip up because I moved.*	How concerned are you about moving by mistake during surgery, causing the doctor to slip up?
Mobility	Walking down the stairs	*I also always have difficulty walking stairs, walking down the stairs. […] Because I cannot see things well, you know. I cannot see depth very well.*	How much difficulty do you have walking down the stairs?
Ocular symptoms	Eye strain	*I have to look in the distance to, well, let my eyes recover from straining, so that they can relax for a while. I do think I have to strain my eyes a lot.*	How often do you experience / How severe is / How much of a problem is eye strain?
Social wellbeing	Describing the floaters	*No, it is, I also noticed, when I explained it to friends, family, when the first floater appeared. Well, you cannot describe it accurately. No, that is very difficult.*	How much of a problem do you have describing the floaters to your friends and family?
Visual symptoms	Vision changing between clear and blurry	*It was like seeing a water drop. And for the entire day, you have some moments when you see clearly, and then this kind of veil appears […]. It is extremely bothersome.*	How often do you experience / How severe is / How much of a problem is your vision changing between clear and blurry?

References

1. Khadka J, Fenwick E, Lamoureux E, Pesudovs K. Methods to Develop the Eye-tem Bank to Measure Ophthalmic Quality of Life. *Optom Vis Sci*. 2016;93(12):1485-1494. doi:10.1097/OPX.0000000000000992

2. Koch F, Deuchler S, Singh P, Müller M, Kohnen T, Schäfer H. Vitrectomy for Vitreous Floaters. *Retinal Physician*. 2015;(12).

3. Ankamah E, Green-Gomez M, Roche W, et al. Dietary Intervention With a Targeted Micronutrient Formulation Reduces the Visual Discomfort Associated With Vitreous Degeneration. *Trans Vis Sci Tech*. 2021;10(12):19. doi:10.1167/tvst.10.12.19

4. Webb B, Webb J, Schroeder M, North C. Prevalence of vitreous floaters in a community sample of smartphone users. *Int J Ophhalmol*. 2013;18(6):402-405. doi:10.3980/j.issn.2222-3959.2013.03.27

5. De Nie KF, Crama N, Tilanus MAD, Klevering BJ, Boon CJF. Pars plana vitrectomy for disturbing primary vitreous floaters: clinical outcome and patient satisfaction. *Graefes Arch Clin Exp Ophthalmol*. 2013;251(5):1373-1382. doi:10.1007/s00417-012-2205-3

6. Tassignon MJ, Ní Dhubhghaill S, Ruiz Hidalgo I, Rozema JJ. Subjective Grading of Subclinical Vitreous Floaters: *Asia-Pacific Journal of Ophthalmology*. 2016;5(2):104-109. doi:10.1097/APO.0000000000000189

7. Schiff WM, Chang S, Mandava N, Barile GR. PARS PLANA VITRECTOMY FOR PERSISTENT, VISUALLY SIGNIFICANT VITREOUS OPACITIES: *Retina*. 2000;20(6):591. doi:10.1097/00006982-200011000-00001

8. Hahn U, Krummenauer F, Ludwig K. 23G pars plana vitrectomy for vitreal floaters: prospective assessment of subjective self-reported visual impairment and surgery-related risks during the course of treatment. *Graefes Arch Clin Exp Ophthalmol*. 2018;256(6):1089-1099. doi:10.1007/s00417-018-3979-8

9. Kim YK, Moon SY, Yim KM, Seong SJ, Hwang JY, Park SP. Psychological Distress in Patients with Symptomatic Vitreous Floaters. *Journal of Ophthalmology*. 2017;2017:1-9. doi:10.1155/2017/3191576

10. Lin T, Li T, Zhang X, et al. The Efficacy and Safety of YAG Laser Vitreolysis for Symptomatic Vitreous Floaters of Complete PVD or Non-PVD. *Ophthalmol Ther*. 2022;11(1):201-214. doi:10.1007/s40123-021-00422-6

11. Osmancevic A. *Treating Vitreous Floaters with ND:YAG Laser Vitreolysis*. School of Medicine, University of Split, Croatia; 2019.

12. Mason JO, Neimkin MG, Mason JO, et al. SAFETY, EFFICACY, AND QUALITY OF LIFE FOLLOWING SUTURELESS VITRECTOMY FOR SYMPTOMATIC VITREOUS FLOATERS. *Retina*. 2014;34(6):1055-1061. doi:10.1097/IAE.0000000000000063

13. Lin J, Su Z, Huang X, Ji X, Yao K. SURGICAL REMOVAL OF DENSE POSTERIOR CAPSULE OPACIFICATION AND VITREOUS FLOATERS IN ADULTS BY POSTERIOR CONTINUOUS CURVILINEAR CAPSULORHEXIS THROUGH THE PARS PLANA AND 23-GAUGE VITRECTOMY. *Retina*. 2016;36(11):2080-2086. doi:10.1097/IAE.0000000000001037

14. Rao RQ, Shah RA, Nausherwan Adil, Akifa Abbas. Efficacy of pars plana vitrectomy (PPV) for visually significant vitreous opacities. *TPMJ*. 2022;29(02):179-182. doi:10.29309/TPMJ/2022.29.02.6720

15. Schulz-Key S, Carlsson JO, Crafoord S. Longterm follow-up of pars plana vitrectomy for vitreous floaters: complications, outcomes and patient satisfaction. *Acta Ophthalmologica*. 2011;89(2):159-165. doi:10.1111/j.1755-3768.2009.01682.x

16. Ludwig GD, Gemelli H, Nunes GM, Serracarbassa PD, Zanotele M. Efficacy and safety of Nd:YAG laser vitreolysis for symptomatic vitreous floaters: A randomized controlled trial. *European Journal of Ophthalmology*. 2021;31(3):909-914. doi:10.1177/1120672120968762

17. Shah CP, Heier JS. Long-Term Follow-Up of Efficacy and Safety of YAG Vitreolysis for Symptomatic Weiss Ring Floaters. *Ophthalmic Surg Lasers Imaging Retina*. 2020;51(2):85-88. doi:10.3928/23258160-20200129-04

18. Delaney YM, Oyinloye A, Benjamin L. Nd:YAG vitreolysis and pars plana vitrectomy: surgical treatment for vitreous floaters. *Eye*. 2002;16(1):21-26. doi:10.1038/sj.eye.6700026

19. Traill A, Verma N, Bylsma G, Curry B. ASSESSMENT OF PATIENT SATISFACTION OF LASER VITREOLYSIS OF PRIMARY VITREOUS FLOATERS. Presented at: The Royal Australian and New Zealand College of Ophthalmologists, 48th Annual Scientific Congress, Melbourne Convention & Exhibition Centre, Melbourne, 19–23 November 2016. https://onlinelibrary.wiley.com/doi/10.1111/ceo.12854

20. Tsai WF, Chen YC, Su CY. Treatment of vitreous floaters with neodymium YAG laser. *Br J Ophthalmol*. 1993;77(8):485-488. doi:10.1136/bjo.77.8.485

21. von Fricken M, Kunjukunju N, Ko G. Floaters: Surgical Management. Presented at: ARVO meeting 2009. https://iovs.arvojournals.org/article.aspx?articleid=2366591

22. Zeydanli EO, Parolini B, Ozdek S, et al. Management of vitreous floaters: an international survey the European VitreoRetinal Society Floaters study report. *Eye (Lond)*. 2020;34(5):825-834. doi:10.1038/s41433-020-0825-0

23. Bessa A. One-year follow-up of patients after yttrium aluminum garnet laser vitreolysis for vitreous floaters. *Egypt Retina J*. 2019;6(1):1-4.

24. Cañote R, Rodríguez D, Izquierdo L, Moncada R, Maldonado C, Henriquez M. Quality of life results after vitreolysis in patients with symptomatic vitreous floaters. 2020;2:2.

25. Lam DSC, Leung HY, Liu S, Radke N, Yuan Y, Lee VYW. Two-Port Pars Plana Anterior and Central Core Vitrectomy (Lam Floaterectomy) in Combination With Phacoemulsification and Intraocular Lens Implantation Under Topical Anesthesia for Patients with Cataract and Significant Floaters: Results of the First 50 Consecutive Cases. *Asia Pac J Ophthalmol (Phila)*. Published online 2017. doi:10.22608/APO.201735

26. Luo J, An X, Kuang Y. Efficacy and safety of yttrium-aluminium garnet (YAG) laser vitreolysis for vitreous floaters. *J Int Med Res*. 2018;46(11):4465-4471. doi:10.1177/0300060518794245

27. Ma J, Huang H, Gao R, Tang S. Ultra-widefield (UWF) Imaging Evaluated the Efficacy of YAG Laser Treatment for Symptomatic Vitreous Floaters. Presented at: ARVO meeting 2019. https://iovs.arvojournals.org/article.aspx?articleid=2746869

28. Nunes GM, Ludwig GD, Gemelli H, Zanotele M, Serracarbassa PD. Long-term evaluation of the efficacy and safety of Nd:YAG laser vitreolysis for symptomatic vitreous foaters. *Arq Bras Oftalmol*. 2022;87(2):0395. doi:10.5935/0004-2749.2021-0395

29. Singh IP. Modern vitreolysis—YAG laser treatment now a real solution for the treatment of symptomatic floaters. *Survey of Ophthalmology*. 2020;65(5):581-588. doi:10.1016/j.survophthal.2020.02.006

30. Souza CE, Lima LH, Nascimento H, Zett C, Belfort R. Objective assessment of YAG laser vitreolysis in patients with symptomatic vitreous floaters. *Int J Retin Vitr*. 2020;6(1):1. doi:10.1186/s40942-019-0205-8

31. Sun X, Tian J, Wang J, Zhang J, Wang Y, Yuan G. Nd:YAG Laser Vitreolysis for Symptomatic Vitreous Floaters: Application of Infrared Fundus Photography in Assessing the Treatment Efficacy. *Journal of Ophthalmology*. 2019;2019:1-7. doi:10.1155/2019/8956952

32. Trujillo-Sanchez GP, Gonzalez-De La Rosa A, Navarro-Partida J, Haro-Morlett L, Altamirano-Vallejo JC, Santos A. Feasibility and safety of vitrectomy under topical anesthesia in an office-based setting. *Indian J Ophthalmol*. 2018;66(8):1136-1140. doi:10.4103/ijo.IJO_289_18

33. Waseem T, DaBreo E, Jiang D, Clawson R, Wagner A, Kapoor K. Pars plana vitrectomy for symptomatic vitreous floaters: Another look. Presented at: ARVO meeting 2019. https://iovs.arvojournals.org/article.aspx?articleid=2744966

34. Woudstra-de Jong JE, Manning-Charalampidou SS, Vingerling JHR, Gerbrandy SJF, Pesudovs K, Busschbach JJ. The impact of vitreous floaters on quality of life: a qualitative study. *J Patient Rep Outcomes*. 2025;9(1):102. doi:10.1186/s41687-025-00934-w
